# A Study on the Use of Milieu Teaching to Promote Overseas Marketers’ Communication Skills and Confidence in Language Learning

**DOI:** 10.3389/fpsyg.2022.923812

**Published:** 2022-06-22

**Authors:** Simeng Jia, Xue Zhao

**Affiliations:** ^1^School of Management, Hebei Finance University, Baoding, China; ^2^School of Marxism, Hebei Finance University, Baoding, China

**Keywords:** milieu teaching, marketers, communication skills, language learning confidence, thinking and behavior

## Abstract

Language plays an extremely important role for people in terms of engaging in various learning activities. Due to the progress of network technologies, it is an immediate goal for enterprises to take a completely new development direction with the application of network technology. Nevertheless, they encounter many difficulties in carrying out overseas marketing such as localization transformation, jet lag, lack of professional marketers, problems with sellers’ product quality, problems with customers’ credit checks, international payment problems, and logistics and delivery problems. These problems mainly result from a difference in language families. The current study was conducted as an experimental study with the participation of overseas marketers in Hebei province. Milieu teaching was implemented with an experimental group and traditional teaching was maintained in the control group for a 20-week (3 h per week) experimental procedure. The research results revealed significantly positive effects of (1) milieu teaching on communication skills, (2) milieu teaching on language learning confidence, and (3) communication skills on language learning confidence. Based on the results, it can be stated that the study is expected to help effectively enhance the communication skills and language learning confidence of overseas marketers to achieve the goals of promoting oral expression and language-use skills.

## Introduction

Language is a conventional symbol system and the common rule or custom in society; it represents concepts by combining symbols and structural and semiotic rules. The human growth process is closely related to cognitive learning, perceptual-motor skills, and social interaction with interaction effect (Alu et al., 2018). Language is also an essential skill in life to express feelings, exchange information, communicate emotions, think, and learn (Ziegler and Hadders-Algra, 2020). In general, humans can learn language from birth without being specifically taught. Crying after birth is a form of communication and learning; crying represents needs, and laughter shows satisfaction or happiness. A 1-year-old toddler can say obscure words, and a 4-year-old child can speak complicated sentences like adults do (Rollins et al., 2019). During the process of growth and learning, language and mental abilities develop together, and language becomes the primary mode of communication that plays an extremely important role in people and in participation in various learning activities. Nevertheless, humans cannot resist the progress of internet technology which continuously presents fast developing products. Therefore, Internet business opportunities are developing positively in the world, so the Internet economic model does not bubble. The application of the Internet is mature, and it is an immediate goal for enterprises to take a completely new development direction with the application of network technology. It is expected that borderless product marketing will be linked by digital transformation so that international trade is not restricted to local time differences. However, there are many difficulties in the conduct of overseas marketing for enterprises such as the transition to a multilingual family, jet lag, lack of professional marketers, production quality of sellers, customers’ credit checks, logistics, and delivery problems. Different language family is a factor in such problems.

Sillera et al. (2018) stated that learning should be done in complete situations; if students are provided rich experiences, such development would be in its best condition. Regarding language development, students need to experience the world to grasp the meanings of relationships and learn a language in the context of the environment under the guidance of important caregivers. Julien and Reichle (2018) pointed out that natural context was the key element in early language development; the interference of environmental language could improve students’ spontaneous and generalized communication skills. They added that with the application of the milieu teaching approach and strategy, students could acquire functional language in a better manner, and that the acquired language could be extended and maintained to improve language ability effectively. Karal and Wolfe (2018) considered that language teaching in natural contexts was the most suitable for language learning; various ordinary, routine, and trivial activities of daily life facilitated simulation and social interaction. In the process, students can express their personal communication needs to demonstrate more communication behaviors and learn and use language more effectively. For this reason, the effect of milieu teaching on overseas marketers’ communication skills and language learning confidence was discussed in this study. The current study is expected to help overseas marketers in terms of improving their communication skills and language learning confidence effectively to achieve the goal of promoting oral expression and language skills.

## Literature Review and Hypothesis

Gauvreau (2019) stated that unlike traditionally controlled situations, instructors in milieu teaching preformulated certain types of skills; milieu teaching focused more on functional communication and emphasized that interference occurs in natural contexts. The major difference from traditional language teaching was that in milieu teaching the lesson was designed to be conducted with discussions rather than memorization of the segment of a skill. In this case, the teaching process was conducted in real-life contexts, addressed the topics that learners have an interest and highlighted communication skills. Hedger et al. (2020) suggested that teaching should focus on students’ motivation and that teaching content should focus on selecting language functionality, practicality, vividness, interactivity, and behavioral reinforcement. Adults interacting with students in natural contexts allowed students to learn new skills and practice skills to receive natural reinforcement of language. Milieu teaching was therefore considered an important strategy to promote the development of students’ communication skills. Dawson Squibb and de Vries (2019) emphasized the teaching process in natural contexts and took functional content as the teaching point to improve students’ spontaneous and generalized communication behavior and learning. Thus, students could improve their communication skills in natural situations. Therefore, the following hypothesis was proposed in this study.

**H1:** Milieu teaching has significant positive effects on communication skills.

Rahn et al. (2019) mentioned that the reflection of language and communication in natural contexts can be seen through learning language use in daily routine and activity. Accordingly, communication should take place in a familiar environment for students to gain confidence in language learning. Similarly, language learning in natural contexts represented language generalization. Turner-Brown et al. (2019) pointed out that milieu teaching is a relatively new language teaching approach. In this approach the teachers use positive interaction in the contexts of social interaction and games. They also follow students’ guidence, opinions, and requests to expand attention and communication. Overall, milieu teaching emphasizes on students’ interests and teachers’ prompts to arouse students’ interest in topics, which in turn promotes students’ confidence in language learning. It is claimed that teaching activities should be conducted in natural contexts and focus on attempts and practice to maintain functional language as well as adopt a teacher–student interaction model. Elek and Page (2019) regarded milieu teaching as the most well-known method for natural language learning in the integrated incidental teaching strategy. The application of incidental teaching is considered the center of the application of milieu teaching. Primary school teachers need to skillfully observe and confirm the timing for teaching. In this case, in order to achieve language learning and communication, it is considered necessary to train in natural environments and respond to students’ concerns. In other words, students’ language acquisition occurs in the natural environment that reduces pressure on students for purposive learning and effectively promotes language learning confidence. Therefore, the following hypothesis was proposed in this study.

**H2:** Milieu teaching has significant positive effects on language learning confidence.

The situations should be designed to help students learn the language as well as enable them to communicate in the target language. An increase in communication behavior can naturally promote students’ communication skills, and, along with improvement in communication skills, a decrease in pressure to communicate naturally improves the language learning confidence. Rogers et al. (2019) mentioned that the situation design strategy in milieu teaching refers to the objective of arousing students’ willingness to communicate actively. Through situation design, the use of the target language becomes a part of students’ daily life and indirectly improves students’ communication skills. In this respect, students’ interests and activities are required in the process of teaching. Besides, adult and peer models should also be provided. Encouraging and responding to students’ progress in communication skills can lead to students improving their confidence in language learning and willingness to communicate. Hancock (2020) mentioned that milieu teaching did not simply teach students to learn a meaningless or unusable language; on the contrary, it provided teaching points to enhance students’ functional language skills and promote the use of a communicative language in an interaction, rather than focusing on language content. In addition to sustained confidence in language learning, the improvement in communication skills can also be seen. Apart from teaching language structure, language function and language learning strategy to improve language proficiency, the milieu teaching strategy highlights generalization ability across contexts more. As a result, the following hypothesis was proposed in this study.

**H3:** Communication skill has significant positive effects on language learning confidence.

## Methodology

### Measurement of Research Variable

#### Communication Skills

Following Hwang et al. (2019), the proportion of spontaneous language for active communication and spontaneous language for passive response in the observation period in all oral utterances (including active/passive spontaneous language and active/passive oral errors) is calculated for communication skills.

#### Language Learning Confidence

Following King et al. (2019), learning confidence contains three dimensions.

##### Thinking and Behavior

This dimension refers to the willingness to make more effort, overcome difficulties, display a high level of perseverance, choose to display challenging behaviors, believe in personal abilities, face difficulties with confidence, and fully develop one’s self.

##### Experience and Observation

This dimension refers to successfully participating in an activity with full understanding and smooth running, and observing the success and failure experiences of people with the same conditions as one’s self.

##### Others, Body, and Mind

Encouragement from others leads to an individual trying harder and accepting challenging activities, and the effects of physiological factors and stressful, nervous, or anxious situations on individual performance.

### Research Participants and Data

In order to achieve the research objective and test the research hypotheses, the experimental design model was adopted. The experiment was conducted for a 20-week (3 h per week) period. The study was conducted with the participation of overseas marketers in Hebei province. Milieu teaching was implemented with an experimental group, and traditional teaching was maintained in the control group. The data were analyzed with SPSS, and the hypotheses were tested by factor analysis, reliability analysis, regression analysis, and analysis of variance.

### Data Analysis

Analysis of variance was conducted to explore the difference between milieu teaching in communication skills and language learning confidence. Additionally, regression analysis was carried out to understand the relationship between communication skills and language learning confidence.

## Results

### Reliability and Validity Analysis

Factor analysis was conducted in this study, and three factors for language learning confidence were extracted, namely “thinking and behavior” (eigenvalue = 2.763, α = 0.9), “experience and observation” (eigenvalue = 2.588, α = 0.92), and “others and body and mind” (eigenvalue = 3.162, α = 0.94). The cumulative covariance explained was found to be 85.271%.

### Effects of Milieu Teaching on Communication Skills and Language Learning Confidence

#### Analysis of Variance of Milieu Teaching in Communication Skills

In Table 1, the results of the analysis of variance are shown to discuss the difference between the teaching models in terms of communication skills. The results showed a significant difference between the teaching models in active communication in terms of communication skills. Milieu teaching (3.89) revealed higher active communication than traditional teaching (3.42). A significant difference between the teaching models was found in passive response in communication skills. Milieu teaching (4.07) showed a higher passive response than traditional teaching (3.55). Accordingly, H1 was supported.

**TABLE 1:** Analysis of variance of milieu teaching in communication skills.

Variable		*F*	*P*	Scheffe *post hoc*
Milieu teaching	Active communication	18.633	0.000*	Milieu teaching (3.89) > traditional teaching (3.42)
	Passive response	24.192	0.000*	Milieu teaching (4.07) > traditional teaching (3.55)

*P < 0.05.

#### Analysis of Variance of Milieu Teaching in Language Learning Confidence

The results of the analysis of variance to discuss the difference between teaching models in terms of language learning confidence are shown in Table 2. The results showed a significant difference between the teaching models in thinking and behavior in terms of language learning confidence. Accordingly, milieu teaching (3.95) was found as enabling higher thinking and behavior than traditional teaching (3.37). The teaching models showed significant differences in experience and observation in language learning confidence. Milieu teaching (4.11) was found to be higher in terms of experience and observation than traditional teaching (3.75). The teaching models revealed significant differences in others and body and mind in terms of language learning confidence. Milieu teaching (4.23) revealed higher others and body and mind compared to traditional teaching (3.69). As a result, H2 was supported.

**TABLE 2:** Analysis of variance of milieu teaching in language learning confidence.

Variable		*F*	*P*	Scheffe *post hoc*
Milieu teaching	Thinking and behavior	20.647	0.000*	Milieu teaching (3.95) > traditional teaching (3.37)
	Experience and observation	27.538	0.000*	Milieu teaching (4.11) > traditional teaching (3.75)
	Others and body and mind	32.392	0.000*	Milieu teaching (4.23) > traditional teaching (3.69)

*P < 0.05.

### Correlation Analysis of Communication Skills and Language Learning Confidence

#### Correlation Analysis of Communication Skills and Thinking and Behavior

A correlation analysis was conducted to test H3. The analysis results are given in Table 3 and reveal significantly positive effects of active communication (β = 2.462**) and passive response (β = 2.238**) on thinking and behavior.

**TABLE 3:** Correlation analysis of communication skills and language learning confidence.

Dependent variable →	Language learning confidence					
Independent variable ↓	Thinking and behavior		Experience and observation		Others and body and mind	
Communication skills	β	*P*	β	*P*	β	*P*
Active communication	2.462**	0.000	2.216**	0.000	2.507**	0.000
Passive response	2.238**	0.000	2.344**	0.000	2.183**	0.000
*F*	27.538		34.175		44.625	
Significance	0.000***		0.000***		0.000***	
*R* ^2^	0.247		0.322		0.381	
Adjusted *R*^2^	0.235		0.307		0.364	

**P < 0.01, ***P < 0.001.Data source: self-organized in this study.

#### Correlation Analysis of Communication Skills and Experience and Observation

The analysis results shown in Table 3 reveal significantly positive effects of active communication (β = 2.216**) and passive response (β = 2.344**) on experience and observation.

### Correlation Analysis of Communication Skills and Others and Body and Mind

The analysis results shown in Table 3 reveal significantly positive effects of active communication (β = 2.507**) and passive response (β = 2.183**) on others and the body and mind. According to the results, it can be stated that H3 was supported.

## Discussion

Changes in environment and design of conditions can increase overseas marketers’ communication opportunities and willingness, learning duration and opportunities, and language learning confidence. For this reason, teachers can arrange for “dissatisfied,” “interested,” “assistance required,” “selection required,” and “unnatural” styles from time to time to increase the opportunities for overseas marketers to communicate with others. Overseas marketers usually have a weaker ability to learn new languages and poor communication and comprehension skills. In this case, language should be introduced in each instruction to combine language and action to assist overseas marketers in terms of language learning. Many overseas marketers show weak expression in the new language, with unclear pronunciation, and are not used to the responses. In this case, simulating responses is the key factor. If overseas marketers get used to simulating responses, it can help in the development of communication skills. Weak generalization ability is a common problem for overseas marketers. In this case, presenting physical objects for communication teaching can avoid difficulties in transformation and promote overseas marketers’ comprehension for effective learning.

## Conclusion

The results of the experimental observation in this study confirm and validate the views of previous research by Julien and Reichle (2018), Dawson Squibb and de Vries (2019), Elek and Page (2019), and Rahn et al. (2019). The findings showed that milieu teaching can improve overseas marketers’ communication skills and increase sentence length and responses to engage them in situations where they can better interact with others. As mentioned by previous researchers, natural situational teaching focuses more on functional communication than traditional teaching and is more like conducting a conversation. Therefore, the teaching takes place in a natural situation, and in the interaction, it can be more involved in how to better interact with others (Gauvreau, 2019). As a consequence, it is safe to claim that milieu teaching can be used for the communication training of overseas marketing professionals. Communication training or language teaching in natural contexts can directly address situations or matters as teaching content in order to eliminate factors that make transferability and generalization difficult. Overseas marketers usually have a poor understanding of a new language. Language teaching, in changing and complicated situations, might be too complicated and difficult for overseas marketers. As discussed by Rahn et al. (2019), the natural situational intervention of language and communication is based on various daily routines and activities to learn how to use the language. Communication needs to be carried out in a familiar environment so that students can feel confident in language learning. For this reason, natural contexts with normal or routine activities in daily life can provide overseas marketers with repeated practice in the real-life environment and gradually increase spontaneous speech. Since overseas marketers have poor communication and comprehension of a new language, they cannot effectively and appropriately respond in many situations. In this case, instructors’ demonstration might easily confuse them. As a result, peers’ or teachers’ drills and demonstrations with immediate reinforcement and feedback can lead overseas marketers to better understand the meanings and promote imitation for learning. Consequently, people’s assistance can be well utilized for teaching.

## Recommendations for Future Research

In addition to communication or language training, natural environment teaching can also be used for other learning activities, such as teaching arithmetic, shopping, and professional skills. Therefore, it is suggested that future research can address different learning activities.

Language learning and communication are extremely complex processes. Children with special needs must acquire language before they can use it. After that, they can show spontaneous communication performance. Therefore, long-term educational intervention can help improve their active and spontaneous communication performance.

The pedagogical intervention in this study was limited to the context of overseas marketers and could not be generalized to other contexts (e.g., home, office, etc.) or objects. Therefore, future research can explore the effects of generalization in different contexts.

## Data Availability Statement

The original contributions presented in this study are included in the article/supplementary material, further inquiries can be directed to the corresponding author.

## Ethics Statement

The present study was conducted in accordance with the recommendations of the Ethics Committee of the Hebei Finance University, and written informed consent was obtained from all the participants. All the participants were asked to read and approved the ethical consent form before participating in the present study. The participants were also asked to follow the guidelines in the form for the research. The research protocol was approved by the Ethical Committee of the Hebei Finance University.

## Author Contributions

SJ performed the initial analyses and wrote the manuscript. XZ assisted in data collection and analysis. Both authors revised and approved the submitted version of the manuscript.

## Conflict of Interest

The authors declare that the research was conducted in the absence of any commercial or financial relationships that could be construed as a potential conflict of interest.

## Publisher’s Note

All claims expressed in this article are solely those of the authors and do not necessarily represent those of their affiliated organizations, or those of the publisher, the editors and the reviewers. Any product that may be evaluated in this article, or claim that may be made by its manufacturer, is not guaranteed or endorsed by the publisher.
